# Exposure to PM_2.5_ and Blood Lead Level in Two Populations in Ulaanbaatar, Mongolia

**DOI:** 10.3390/ijerph13020214

**Published:** 2016-02-15

**Authors:** Undarmaa Enkhbat, Ana M. Rule, Carol Resnick, Chimedsuren Ochir, Purevdorj Olkhanud, D’Ann L. Williams

**Affiliations:** 1 School of Public Health, Mongolian National University of Medical Sciences, Ulaanbaatar-14210, Mongolia; chimedsuren@mnums.edu.mn (C.O.); purevdorj@mnums.edu.mn (P.O.); 2 Department of Environmental Health Sciences, Johns Hopkins Bloomberg School of Public Health, Baltimore, MD 21205, USA; arule1@jhu.edu (A.M.R.); cresnic2@jhu.edu (C.R.); dwilli20@jhu.edu (D.L.W.)

**Keywords:** Air pollution, indoor Air, particulate matter, PM2.5, blood lead, cookstove, Mongolia, coal burning, women’s health, exposure assessment

## Abstract

Approximately 60% of the households in Ulaanbaatar live in gers (a traditional Mongolian dwelling) in districts outside the legal limits of the city, without access to basic infrastructure, such as water, sewage systems, central heating, and paved roads, in contrast to apartment residents. This stark difference in living conditions creates different public health challenges for Ulaanbaatar residents. Through this research study we aim to test our hypothesis that women living in gers burning coal in traditional stoves for cooking and heating during the winter are exposed to higher concentrations of airborne PM_2.5_ than women living in apartments in Ulaanbaatar, Mongolia, and this exposure may include exposures to lead in coal with effects on blood lead levels. This cross-sectional study recruited a total of 50 women, 40–60 years of age, from these two settings. Air sampling was carried out during peak cooking and heating times, 5:00 p.m.–11:00 p.m., using a direct-reading instrument (TSI SidePak™) and integrated polytetrafluoroethylene (PTFE) filters using the SKC Personal Environmental Monitor. Blood lead level (BLL) was measured using a LeadCare II rapid field test method. In our study population, measured PM_2.5_ geometric mean (GM) concentrations using the SidePak™ in the apartment group was 31.5 (95% CI:17–99) μg/m^3^, and 100 (95% CI: 67–187) μg/m^3^ in ger households (*p* < 0.001). The GM integrated gravimetric PM_2.5_ concentrations in the apartment group were 52.8 (95% CI: 39–297) μg/m^3^ and 127.8 (95% CI: 86–190) μg/m^3^ in ger households (*p* = 0.004). The correlation coefficient for the SidePak™ PM_2.5_ concentrations and filter based PM_2.5_ concentrations was r = 0.72 (*p* < 0.001). Blood Lead Levels were not statistically significant different between apartment residents and ger residents (*p* = 0.15). The BLL is statistically significant different (*p* = 0.01) when stratified by length of exposures outside of the home. This statistically significant difference in increased BLL could be due to occupational or frequent exposure to other sources of indoor or outdoor air pollution that were not measured. Blood lead levels from our study population are the first study measurements published on women aged 40–60 years of age in Mongolia.

## 1. Introduction

The World Health Organization, (WHO) has ranked Ulaanbaatar as the second most polluted city in the world, and a great deal of attention has been given to the issue of outdoor air pollution [1,2,3]. Air pollution is considered to be a route of exposure to heavy metals, such as lead (Pb) in the general population of Ulaanbaatar during heating periods due to the extensive use of coal for energy production and heating sources [4]. The World Bank reports that particulate matter is one of the main air pollutants in Ulaanbaatar, due to emissions originating from domestic heating and cooking needs, increasing number of vehicles, growing number of industries, construction activities, and higher electricity demand [1,2]. It remains unclear if air pollution is a contributor to lead exposures in Ulaanbaatar. This highlights the public health significance of investigating indoor contributions to air pollutants to assess exposures.

There has been a significant increase in ger (traditional Mongolian dwelling) residents due to the influx of populations to Ulaanbaatar in the last decade; many migrants are herders who lost their domestic animals to harsh winter climates, and without other economic opportunities they have moved to Ulaanbaatar seeking work and better lives [5]. As ger migrants move into Ulaanbaatar they create new districts outside the legal boundaries of the city. Currently, there are approximately 175,000 ger households, 60% of the total city residents living in ger districts do not have access to basic infrastructure such as water, sewage systems, central heating, and paved roads [6,7].

Inhalation exposures associated with high concentrations of airborne particulate matter (PM) have long been associated with negative health effects [8,9]. Fine particulate matter, PM_2.5_ a regulated pollutant by the US EPA, describes particles less than or equal to 2.5 microns (μm) in diameter, that can be deposited deep into the respiratory system and can cause severe health problems including chronic obstructive pulmonary diseases (COPD), lung cancer, and cardiovascular disease [2,10]. A study conducted by Allen *et al.* (2013) estimated that 29% of cardiopulmonary deaths and 40% of lung cancer deaths in Ulaanbaatar are attributable to air pollution which accounts for 10% of all-cause mortality rate in the city [11]. The World Bank Report indicates the worst annual average concentration of PM_2.5_ in Ulaanbaatar is 25 times higher than the Mongolian Air Quality Standard of 25 μg/m^3^ (MNS 4585/2011) and 17–18 times higher than the World Health Organization interim ambient air quality targets for developing countries of 35 μg/m^3^ [1]. Measurements at 37 urban and residential areas in Ulaanbaatar collected between June 2009 and May 2010 have reported daily mean PM_2.5_ concentration of 22.8 ± 9.0 μg/m^3^ in the summer and 147.8 ± 61.2 μg/m^3^ in the winter [11]. Measured winter PM_2.5_ in Ulaanbaatar can reach peaks up to 750 μg/m^3^ [1,2].

In Ulaanbaatar, people are at higher risk of indoor air pollution during winter months when they tend to spend more time indoor due to colder weather when temperatures can drop to −40 °C and there are shorter daylight hours. Ger households consist of an expandable wooden circular frame that is covered with several layers of felt wool for insulation, with an upper opening for exhaust of cooking and heating stoves. The gers do not have windows and indoor environments characteristically have limited air circulation and poor ventilation. Indoor air pollution is mainly generated through stove leakage, through the process of ash removal, or through the fire starting process. Another contributor to indoor air pollution is from “neighborhood” pollution defined by indoor-outdoor pollution differences; even when indoor coal smoke is dispersed outside, high levels of local pollution can re-enter the household. In particular this is the case for cold winter days with poor atmospheric dispersion [12]. Studies that have explored contributions of indoor and outdoor PM in the US found that the best estimate of the mean contribution of outdoor PM_2.5_ to the indoor mass concentration was 73% and to personal exposure was 26% [13]. However, air exchange rate was found to be an important determinant of indoor air pollution levels, and there is no data on air exchange rates in Mongolian gers or apartments. Considering these factors, indoor air pollution could be a major route of exposure to PM_2.5_.

Lead concentrations in whole blood are considered to be the primary biomarker used to monitor human exposure to lead (Pb) from ongoing or recent exposures [14]. Toxicokinetic studies have shown that lead has a mean biological half-life in blood of about 30–40 days in adults [14]. The US Centers for Disease Control and Prevention (CDC) has reduced the level of concern for BLL from 10 μg/dL (0.48 μmol/L) to 5 μg/dL (0.24 μmol/L) as recent studies have shown possible adverse health effects at BLL less than 10 μg/dL in children [15]. This suggests that there is no safe level of exposure [14]. Exposure to lead occurs through a combination of inhalation and oral exposures [16]. Mongolia has estimated the level of heavy metals in the largest coal mining sites and the result shows that lead concentration in coal is estimated to be 35.95 mg/kg in the Baganuur mine, the largest source of coal for Ulaanbaatar [17]. Due to the high level of air pollution in Ulaanbaatar from the extensive use of coal, and documented lead content in coal mining sites, it is critical to understand potential lead exposure through the inhalation pathway. Therefore, our goal was to test if these high levels of air pollution contribute to blood lead concentrations in a subset of Ulaanbaatar residents.

Two studies were found that measured BLLs in Mongolia and were studies of children [17,18]. In 2005, Burmaa *et al.*, reported that average BLLs among children 7–14 years old were 16.54 ± 9.50 μg/dL [17]. The study among six- to eight-year-old school children conducted by Praamsma *et al.*, in 2014, reported that geometric mean BLLs were 5.3 (95%: 4.9-5.7) μg/dL showing a decrease in BLL measurements of children in Ulaanbaatar [18]. This decrease could be due to a ban on leaded gasoline in 2008 [19,20].

On a global scale, there have been numerous studies conducted on cookstoves and indoor air pollution, as well as BLL, among older women and the general population. Of relevance were studies conducted by McCracken *et al.*, (2013) and Zhang and Smith (2007). These studies demonstrate that biomass and coal use increase the risk of various health problems, and PM_2.5_ and CO are good indicators for poor indoor air quality. However, none of the cookstove studies looked at blood lead since most cookstoves use biomass fuels that do not contain lead. This highlights the need for conducting a study to investigate indoor air pollution and BLLs among women who use coal for cooking and heating and women who do not. It is expected that stove use would add to the daily outdoor exposure as 80% of air pollution is contributed from coal burning in ger districts across the city [1]. Ultimately, through this research our aim was to test our hypothesis of whether women living in gers who use coal for cooking and heating during the winter are exposed to higher concentrations of PM_2.5_ and have higher BLL than women living in apartments in Ulaanbaatar, Mongolia.

## 2. Experimental Section

### 2.1. Materials and Methods

#### 2.1.1. Study Design

The goal of this cross-sectional study was to assess exposure to PM_2.5_ and BLL among ger residents and apartment residents in Ulaanbaatar Mongolia. Air sampling was conducted at both sites, one time during peak cooking and heating times, from 5:00 p.m. to 11:00 p.m. Both groups were sampled during the same time period in January and February 2015, when PM exposures are likely to be at their maximum for both groups.

#### 2.1.2. Study Population

Our targeted population was women of age 40–60 from Ulaanbaatar, Mongolia. Women of this age were suspected to have long-term and high exposure to PM_2.5_ due to cooking and managing the heating of the house/ger as part of their daily chores, and are therefore at high risk of exposure to contaminants from coal burning. Two groups of 25 participants from these two different residential settings were recruited for the study. The “ger” group was 25 women living in gers in the “Doloon Buudal” area who used coal for cooking and heating utilizing a traditional stove, which consists of the combustion box and a central chimney that extends outside the roof of the ger. The “apartment” group was comprised of 25 women living in apartment complexes in the “Zaisan Hill” area of Ulaanbaatar who use electric stoves for cooking. Exclusion criteria were women who smoke, women residing in homes where tires and trash were used for fuel, and women who had moved during the last year. Exclusions due to type of occupation or other exposures were not considered.

### 2.2. Indoor Air Quality Measurement

Both a direct reading method and a gravimetric filter method were used to compare the sampling methods for determining indoor air pollution [21]. An integrated sample was obtained using 37 mm polytetrafluoroethylene (PTFE) membrane filters with a 2-micron pore size (R2PJ037 SKC Inc., Ann Arbor, MI, USA) placed inside a SKC Personal Environmental Monitor (PEM 761-203B, SKC Inc., Eighty Four, PA, USA) with a cut size of 2.5-micron. These PEMs were connected to AirChek vacuum pumps (SKC 224-44XR, SKC Inc., Eighty Four, PA, USA) for gravimetric sampling and metals analysis. An electronic flow calibrator (BIOS Defender 510, MesaLabs Inc, Butler, NJ, USA), was used to calibrate the vacuum pumps at a flow rate of 2 L/min ± 0.10 L/min and the flow rate was validated post-sampling. All PTFE filters were pre- and post-weighed in a temperature and humidity controlled room using EPA FRM standard operating procedures [22]. The integrated gravimetric PM_2.5_ mass concentration was calculated based on the sampling average flow rate, elapsed time, pre- and post-weight and a blank correction factor.

Real-time concentrations of indoor ambient PM_2.5_ were measured using the TSI SidePak™ (SP330, TSI Inc., Shoreview, MN, USA). The SidePak™ determines the real-time particle mass concentration by a light scattering technique. A 2.5-micron cut size impactor inlet was used for size selection. The SidePak™ was calibrated at the flow rate of 1.7 L/min and zeroed prior to each sample according to manufacturer specifications. Average PM_2.5_ concentration was logged every minute with an average measurement taken every 10 seconds. The time averaged PM_2.5_ mass concentration was calculated based on the minute interval concentration over the sampling duration.

Since the light-scattering properties of PM_2.5_ vary substantially with particle size and composition, and SidePaks are calibrated using Arizona road dust, light-scattering PM_2.5_ measurements must be calibrated to the specific aerosol being sampled. We determined a calibration factor based on our co-located gravimetric PM measurements to correct the SidePak measurements to evaluate coal burning smoke in a relevant setting. The coal specific calibration factor for the PM_2.5_ SidePak was estimated by regressing gravimetric PM_2.5_ on arithmetic mean real-time PM_2.5_ concentrations. The estimated regression coefficient, 0.34 was then used as a calibration factor and applied to the SidePak PM_2.5_ data.

Nicotine samples were collected using a passive method to evaluate incidental contribution to PM concentrations and potential exposure to second-hand smoke [23]. Second-hand smoke has been shown to be a major contributor to higher indoor air PM concentrations and metals exposure in homes [24,25]. The secondhand smoke was collected using a filter badge treated with sodium bisulfate, which was prepared at Johns Hopkins Bloomberg School of Public Health (JHBSPH) and was shipped to Ulaanbaatar prior to the research study. Ten-percent blanks and duplicates were collected during sampling. Upon completion of the study, badges were shipped to JHBSPH. Filters were extracted with an internal standard (isoquinoline, Sigma-Aldrich, St. Louis, MO, USA) and analyzed using a gas chromatograph with a nitrogen phosphorus detector (GC-FTD, Shimadzu GC-2014, Shimadzu, Columbia, MD, USA). Nicotine was separated using a capillary column (SHRXI-5MS, Shimadzu, Columbia, MD, USA). The limit of detection (LOD) for nicotine analysis was 0.74 μg/m^3^ and from the laboratory criteria all samples below the LOD were reported as LOD/square root of 2 or 0.52 μg/m^3^.

All the air sampling equipment was placed approximately one meter away from the traditional stoves in the ger and electric stoves in apartments and were elevated to approximately 1.5 meters off the floor to reflect exposures in the breathing zone.

### 2.3. Sampling Quality Control

The quality control procedure included pre- and post-calibration of sampling devices (the AirChek sampling pump at a flow rate of 2 L/min ± 0.10 L/min), and the use of field blanks and duplicate samples for PM_2.5_ measurement and nicotine passive monitoring. For gravimetric analysis there were 10% field blanks (4.9 ug/m^3^) and a relative percent difference for the 10% duplicate samples was 22%. Participants were randomly chosen for duplicate sampling. Duplicate sample monitors were placed side-by-side in order to capture the same sampling microenvironment. All filters along with field blanks and duplicates were tightly sealed and stored in plastic bags and stored in a non-humid place both before and after sampling. The filters and nicotine monitors were transported from Ulaanbaatar to the laboratory at Johns Hopkins Bloomberg School of Public Health in Baltimore three weeks after the collection of samples.

### 2.4. Measurement of BLLs

Blood lead levels were measured using a Clinical Laboratory Improvement Amendment (CLIA) waived validated test, the Lead Care II©, (ESA Biosciences, Inc., Chelmsford, MA, USA). The Lead Care II© has been certified for use by the CDC Multi-Element Proficiency Program (LAMP). According to Lead Care II© protocols, hands were washed and air-dried to prevent external contamination of the blood sample. Approximately 50 μL (approximately 2 drops) of whole blood was collected from the participant’s fingertip using a capillary tube provided in the test kit. During blood collection procedures, the researcher cleaned her hands with antiseptic soap and wore gloves for personal protection and to prevent the introduction of external contaminants. The manufacturer’s instrumental detection limit of the LeadCare II© is 3.3 μg/dL and the instrument will report values up to 65 μg/dL. For BLL samples that were below the LOD, LOD/ square root of 2 or 2.3 μg/dL was used for statistical analysis. This replacement method is considered to be a good estimate for highly censored data [26].

### 2.5. Questionnaires

A household inspection based on researcher observations of general housing status, living conditions, type of ventilation system, general cleanness of the house, and presence of smoke in the house was collected from all participants by study staff. A general health/exposure related questionnaire was administered to assess the health status of the participants that addressed presence of any chronic disease or symptoms related to high levels of lead exposure. Employment status, average amount of time spent at home, possible sources of lead exposures, such as drinking water, and high-risk occupations, such as miners and battery industry workers were also evaluated. Apartment residents were asked PM exposure related questions such as the, presence of an exhaust hood in the kitchen, and frequency of the window opening.

### 2.6. Statistical Analysis

Statistical analyses were performed using STATA 11 (StataCorp, College station, TX, USA). Descriptive statistics were generated to describe the characteristics of the participants including occupation, age distribution, cooking frequency, chronic disease prevalence, average amount of time spent at home and any symptoms reported by participants. Air concentrations of PM_2.5_ and BLL were quantified in total and stratified by residence type. Because measured ambient PM has a positively skewed distribution, the data was converted to a log scale. A two sample t-test was used to analyze the geometric mean value of PM_2.5_ between groups. Blood lead levels were also positively skewed due to a high number of non-detected samples. Analysis of BLL based on house type and duration of stay at homes were run using a t-test and a Chi-squared test for detected and undetected values. The results of both of these analyses were consistent with no statistically significant differences observed between house types. We estimated a coal dust-specific calibration factor for PM_2.5_ SidePak measurements by regressing the gravimetric PM_2.5_ onto the arithmetic mean value of the SidePak PM_2.5_ concentrations. The estimated regression coefficient, 0.34 (CI: 0.26–0.41) (*p* < 0.001), was then used as a calibration factor and applied to the SidePak PM_2.5_ data before log transformations and analysis.

### 2.7. Ethical Considerations

The research plan, recruitment procedure and consent forms were approved by the Johns Hopkins Bloomberg School of Public Health Institutional Review Boards IRB000006045 and the Ethics Committee of Mongolian National University of Medical Sciences #14-14/1A. To protect participant confidentiality, participants were assigned a participant ID number, all paperwork included only the participant ID, number, age and no other identifying information. Written consent forms were signed by the participant prior to air sampling and fingerstick blood collection.

## 3. Results

A summary of participant occupational status, participant age, exposure and health related questions stratified by residency type are presented in Table 1 (100% response). Overall, 21 (42%) participants were unemployed or retired, while 29 (58%) were employed full time. The average age of the participants was 51.5 ± 5.9 and there was no statistically significant difference for the age range among people living in apartments and people living in gers (*p* = 0.85). We defined chronic disease as any present long-term illness such as diabetes, cirrhosis and hypertension. Chronic disease prevalence was collected based on the participants self-report. There were no statistically significant differences in chronic disease prevalence between apartment residents (52%) and ger residents (56%) (*p* = 0.34).

**Table 1:** Summary statistics of exposure assessment and health related questions stratified by residence type.

Type of Housing		Apartment (*n* = 25)	Ger (*n* = 25)	*p*-Value *
		Frequency (%)	Frequency (%)	
Occupation (*N* = 50)	Stay at home	9 (36)	12 (48)	
	Employed full time	16 (64)	13 (52)	
Participant age		51.4 ± 6	51.6 ± 6	0.85
Chronic disease prevalence ^†^	Yes	13 (52)	14 (56)	0.34
	No	12 (48)	11 (44)	
Cooking frequency in the last 48 h	None	7 (28)	1 (4)	
	Less than 3 times	7 (28)	6 (24)	
	More than 3 times	11 (44)	18 (72)	0.02
Time spent at home (hours)	Less than 10 h a day	4 (16)	1 (4)	
	10–15 h a day	8 (32)	10 (40)	
	15–20 h a day	7 (28)	3 (12)	
	20 or more hours a day	6 (24)	11 (44)	

^†^ Diabetes, cirrhosis, hypertension arthritis and atherosclerosis; * *p*-value is compared between groups by Chi-squared test.

People living in gers cooked more often than people living in apartments (24 *vs.* 18) (*p* = 0.02) Seven participants (28%), of apartment residences had no cooking activity during the last 48 hours due to full-time employment. Among all 50 participants, regardless of house type, 19 participants (38%) had cooked four times in the previous 48 hours, with 18 (72%) participants residing in gers.

Duration of exposure was based on participant report of the time they spent in their homes. Among all study participants regardless of house type, 18 participants (36%) spent 10–15 hours at home, 17 participants (34%) spent 20 or more hours at home, 10 participants (20%) spent 15–20 hours at home, and 5 participants (10%) spent 10 or less hours a day at home.

Summary statistics of PM_2.5_ concentration and nicotine concentration stratified by housing type are presented in Table 2.

**Table 2:** Measured ambient PM, air nicotine among participants stratified by residence type.

Measurement Type	Apartment		Ger		*p*-Value *
	Geometric Mean (95% CI)	Sample (*n*)	Geometric Mean (95% CI)	Sample (*n*)	
**Real-time PM_2.5_ concentration (μg/m^3^)**	31.5 (95% CI: 17–99)	25	100 (95% CI: 67–187)	(25)	<0.001
**Filter based PM_2.5_ concentration (μg/m^3^)**	52.8 (95% CI: 39–297)	23	127.8 (95% CI: 86–190)	(25)	0.004
**Nicotine concentration (μg/m^3^)**	0.5 (95% CI: 0.3–0.5)	25	0.6 (95% CI: 0.3–0.7)	(24)	0.48

* *p*-value is compared between groups by 2 sample t-test.

**Figure 1:**
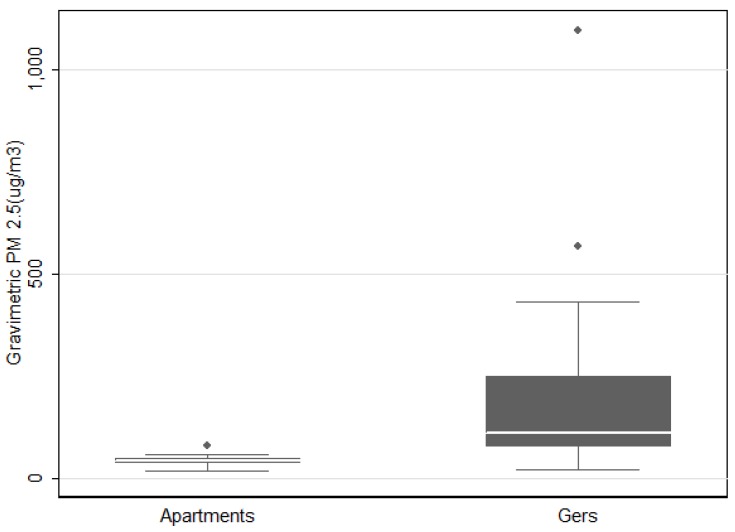
Concentration of PM_2.5_ using gravimetric method (excluding 2 outliers).

The GM of the SidePak™ PM_2.5_ concentration in the apartment group was 31.5 (95%CI: 17–99) μg/m^3^ while in the ger group the GM concentration was 100 (95%CI: 67–187) μg/m^3^ (*p* < 0.001). The GM of filter based PM_2.5_ concentrations (Figure 1) in apartments were 52.8 (95%CI: 39–297) μg/m^3^ and 127.8 (95%CI: 86–190) μg/m^3^ in ger households (*p* = 0.004). Two outlier points were eliminated from the filter based data analysis because the samples were accidently dropped during field collection.

Nicotine concentration was measured in both groups. All 25 samples from the apartment group had nicotine concentrations below the LOD. In the ger group 23 samples were < LOD with one home measuring 3.76 μg/ m^3^. One monitor was not able to be recovered after sample collection.

**Figure 2:**
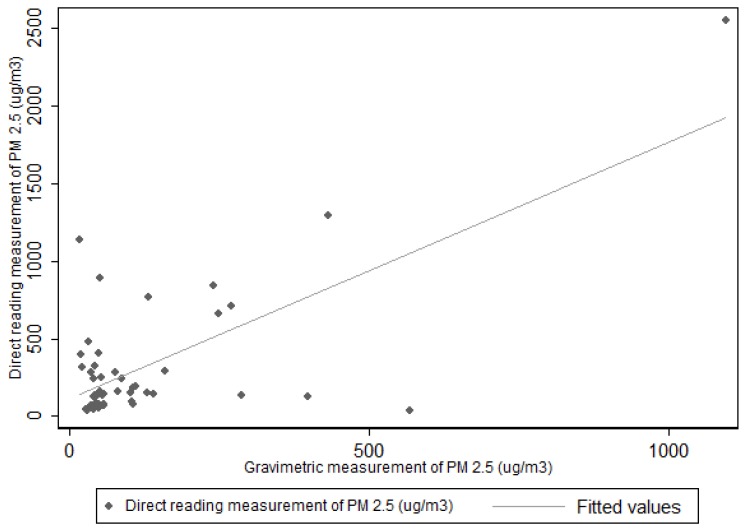
Correlation between PM_2.5_ gravimetric method and direct reading method using SidePak™ (r = 0.72).

The US EPA has established the gravimetric sampling method as the reference method for the determining PM_2.5_ in ambient air, and the filter based samples are considered as the “gold standard” [27]. The GM of all PM_2.5_ samples measured using the SidePak™ (50) were correlated with 48 PM_2.5_ gravimetric samples. The Spearman rank correlation coefficient (Figure 2) for the SidePak™ PM_2.5_ concentration and gravimetric PM_2.5_ concentration was (r = 0.72) with (*p* < 0.001).

The BLL of participants were categorized as detected and non-detected values based on house type and length of exposure. There was no statistically significant difference in number of detected BLL among apartment residents and ger residents (*p* = 0.15). Overall, 29 samples (58%) of all BLL samples were below the LOD of the instrument.

We also stratified BLL status by detected and non-detected values based on length of exposure at home (Table 3). We compared those participants who spend 15 or more hours at home to those who spend less than 15 hours a day at home. Blood lead levels were found to be statistically significantly different among apartment residents and ger residents based on time they spent outside of the home (*p* = 0.01).

**Table 3:** Number of Blood Lead Level samples stratified by detected and non-detected values based on housing type and length of exposure at home.

Exposure Factors	Group	Number of Detected Sample *n* (%)	Number of Non-detected Sample *n* (%)	*p*-Value *
**Housing type (*N* = 50)**	**Apartment**	8 (16)	17 (34)	
	**Ger**	13 (26)	12 (24)	0.15
**Length of exposure at home (*N* = 50)**	**>15 h**	7 (14)	20 (40)	
	**≤15 h**	14 (28)	9 (18)	0.01

* *p*-value is compared between groups by Chi-squared test.

## 4. Discussion

Our study sought to identify PM and potential lead exposure in two groups of adult Mongolian women based on home type and differential airborne exposures in Ulaanbaatar. The hypothesis of the study was that indoor air pollution is higher in ger households than in apartment buildings, and that exposure to PM due to coal combustion is associated with lead exposure among adult women.

This study identified that women ages 40–60 years who live in traditional ger housing are exposed to higher concentrations of PM_2.5_ than women who live in apartments in Ulaanbaatar, Mongolia. Of concern, mean PM_2.5_ concentrations (127.8 (95% CI: 86–190)) in ger households were two to three times higher than the Mongolian Air Quality Standard of 50 μg/m^3^ for a 24 hour average. Furthermore, there was a statistically significant difference in PM_2.5_ concentration between apartments and gers (*p* < 0.001). This result is inconsistent with an indoor air quality study conducted by Enkhtsetseg *et al.*, (2007) where there was no statistically significant difference observed between apartments and gers when coal was used for combustion (*p* = 0.44) [28]. The PM_2.5_ was measured using a gravimetric method (the gold standard method); however, that study had several limitations including the small sample size of 10 ger households and five apartments. The sampling took place in the months of March and April, considered to be relatively warm, where heating activity is significantly reduced which may explain the lack of significant findings. Numerous scientific publications indicate the health effects of PM_2.5_ and cardiovascular disease-related mortality and morbidity [8,11], therefore, the high exposure to PM_2.5_ continues to be an environmental risk factor for cardiovascular disease, the leading cause of morbidity and mortality in Mongolia.

This study is the first to measure BLLs among an older female adult population in Mongolia. The GM BLL of our study population (3.1 μg/dL) was two times higher than the general population in the United States, 1.38 μg/dL (NHANES 2007–2008) (CDC, 2011) [28]. In comparison, a study of the blood lead concentrations, 2009–2010, of the general female population in China has been reported as 3.04 μg/dL [26], similar to our study population. In Japan, the average BLL in women was measured to be 1.58 μg/dL (collected in 2000) [28]. There is limited information on lead levels in Mongolian coal and our study results show no statistically significant difference in BLL observed between women living in ger households and apartments. These results suggest that indoor air pollution to PM_2.5_ may not be the primary source of exposure to lead considering the significant difference observed in PM_2.5_ concentrations between the two groups.

In our study we found that there is a statistically significant difference in BLL between women who spend more time at home and women who spend less time at home. As exposure is characterized by the magnitude, frequency and duration of contact with the agent, we found that duration is an important factor in our exposure assessment. Further analysis of our results show three women with the highest BLLs of 6.1 μg/dL, 6.3 μg/dL, and 5.8 μg/dL all lived in apartments with average measured PM_2.5_ concentrations of 42 μg/m^3^, 43 μg/m^3^, and 57 μg/m^3^, respectively. These three participants were also 50, 55 and 57 years of age. Individual differences in metabolism during menopause could explain these observations as accumulated lead from long term exposure can be reabsorbed into the circulatory system through the bone demineralization process [29]. Unfortunately we did not conduct a clinical evaluation of osteoporosis to explore this further. Furthermore, all three participants were employed full-time, one as a housekeeper at an electric company office, another as a high school art teacher, and one as an accountant at a mining equipment company. They have maintained the same jobs for the last 12, 31 and 9 years, respectively. This higher level of BLL among apartment residents eliminates coal burning for home heating as a potential source of exposure. This raises another possibility of greater exposure to lead in occupational settings or increased exposure among study participants to other sources of lead.

One of the limitations of our study is that we only measured PM_2.5_ concentrations inside participant homes with no additional monitoring of PM_2.5_ where participants spent the remaining time during their day. This increases our uncertainty when trying to determine actual source exposures to lead from airborne PM_2.5_. In the future, studies monitoring occupational settings and other microenvironments should be conducted in order to investigate factors that may contribute to lead exposures. An additional limitation is that we did not assess lead associated with BMI, dietary intake or alcohol consumption which may be affected by differential daily exposures not associated with airborne lead exposures in these two populations.

Historically one of the primary sources of airborne lead exposure was from the use of leaded gasoline. Lower BLL measured in our study population and in previous studies suggests there has been a decline in BLL that may likely be due to the government ban on leaded gasoline in 2008 [19,20].

Even though the LeadCare II is a valid rapid testing method, the instrumental LOD of 3.3μg/dL, makes it impossible to quantify levels of blood lead concentrations that fall below this limit. In our study, 29 of 50 (58%) blood lead samples were below the limit of detection. Of 29 undetected samples 17 samples were from apartment residents (68%) and 12 samples were from ger residents (48%).

To the best of our knowledge, there has been only one published scientific study on PM_2.5_ and indoor air pollution in Mongolia [27]. Results from that study indicate the mean PM_2.5_ concentrations in ger households were 55 μg/m^3^ compared to 43 μg/m^3^ (*p* = 0.44) in apartments while in our study PM concentrations are two to three times higher than this previous study. Our study results add to the scientific information on PM_2.5_, BLL and indoor air pollution, and can serve as comparison values for further studies in Mongolia.

## 5. Conclusions

Our study provides supporting evidence that women and other individuals living in ger households are exposed to significantly higher concentrations of PM_2.5_ than people living in apartments in Ulaanbaatar Mongolia. However, a difference was not observed in BLL measurements for older women living in the two settings, and we found overall low levels of BLL. There is a statistically significant difference between BLLs in women who spend less than 15 h a day at home regardless of housing type. These differences could be due to exposures to lead in occupational settings or frequent exposure to other sources of lead. Our study was the first to evaluate BLLs in an adult population in Mongolia. This study contributes to the feasibility and evaluation of methods to collect PM in Ulaanbaatar and provides baseline data for the development of more extensive studies in Mongolia to evaluate PM, PM constituents and PM related biomarkers. Our results suggest that elevated BLLs may not be a major public health issue for older adult women in Ulaanbaatar. However, the elevated PM_2.5_ levels observed in Ulaanbaatar, as well as those observed inside ger homes are concerning and continue to be a significant risk to public health.
